# Comparative Analysis of G1P[8] Rotaviruses Identified Prior to Vaccine Implementation in Pakistan With Rotarix™ and RotaTeq™ Vaccine Strains

**DOI:** 10.3389/fimmu.2020.562282

**Published:** 2020-10-02

**Authors:** Asma Sadiq, Nazish Bostan

**Affiliations:** Department of Biosciences, COMSATS University (CUI), Islamabad, Pakistan

**Keywords:** G1P[8], rotavirus, genotype, antigenic epitopes, emergence

## Abstract

Group A rotavirus (RVA) is the leading cause of severe childhood diarrhea globally, even with all effective interventions, particularly in developing countries. Among the diverse genotypes of RVA, G1P[8] is a common genotype that has continued to pervade around the world, including Pakistan. Two universally accepted rotavirus vaccines-Rotarix™ and RotaTeq™ contain the genotype G1P[8]. The current work was aimed at identifying differences between antigenic epitopes of Pakistan’s G1P[8] strains and those of the two licensed vaccines. We sequenced 6 G1P[8] rotavirus strains previously reported in Rawalpindi, Islamabad, Pakistan in 2015 and 2016 for their outer capsid genes (VP7 and VP4). Phylogenetic analysis was then conducted in order to classify their specific lineages and to detect their association with strains isolated throughout world. Compared with the Rotarix™ and RotaTeq™ vaccine strains (G1-lineage II, P[8]-lineage III), our study G1-lineage I, P[8]-lineage IV strains showed 3 and 5 variations in the VP7 epitopes, respectively, and 13 and 11 variations in the VP4 epitopes, respectively. The G1 lineage II strains showed no single amino acid change compared to Rotarix™ (lineage II), but exhibited changes at 2 positions compared to RotaTeq™ (lineage III). So, this has been proposed that these G1 strains exist in our natural setting, or that they may have been introduced in Pakistan from other countries of the world. The distinct P[8]-lineage IV (OP354-like) strains showed twelve and thirteen amino acid variations, with Rotarix™ and RotaTeq™ (lineages II and III) strains, respectively. Such findings have shown that the VP4-P[8] component of the G1P[8] strains circulating in Pakistan differs considerably from that of the vaccine viruses compared to that of the VP7-G1. To monitor the long-term effects of vaccines on the emergence of G1P[8] strains with different lineages, routine and successful monitoring of these strains will be crucial.

## Introduction

Rotavirus-related gastroenteritis (RVGE) is a significant global health concern contributing to 128,500 deaths among children younger than 5 years worldwide in 2016, as of the most recent data (1). While the rate of diarrhea-associated mortality has decreased globally over the last decade, the burden of rotavirus diarrhea remains significant in low-income settings (2). According to estimates from the WHO, approximately half of all rotavirus-associated deaths occurred in India, Nigeria, Pakistan and the Democratic Republic of Congo in 2013 (3). For an estimated 393,959 deaths under five years of age in children in Pakistan, 14,700 are due to rotavirus diarrhea (4). Pakistan is in its early stages of using rotavirus vaccines to combat childhood rotavirus infections (5).

Rotaviruses in the family *Reoviridae* are icosahedral and triple layered viruses with eleven segmented double- stranded RNA (ds-RNA) genome (6, 7). All eleven segments express six structural (VP1-VP4, VP6 and VP7) and six non-structural proteins (NSP1-NSP6) (8). Based on genes coding for the two outer capsid proteins (VP7 and VP4), RVAs are categorized into G (glycoprotein) and P (protease-sensitive) genotypes. Till now, 36 genotypes of G and 51 P have been reported worldwide (9). The epidemiological studies indicate that at least six human RVA genotypes G1P[8], G2P[4], G3P[8], G4P[8], G9P[8], and G12P[8] are globally prevalent in human infections (10).

Rotavirus is a segmented virus that is continuously evolving (11). There is substantial variation in the distribution of rotavirus genotypes globally. The most prevalent strains that cause serious disease change year after year within and between country to country. The error-prone RNA-dependent polymerase, without proofreading ability, offers various evolutionary mechanisms for rotavirus, involving point mutation, reassortment, recombination, and interspecies transmission (12, 13).

The battle against rotavirus infection has been a big concern for WHO (World Health Organization) for many years (14). Four WHO pre-qualified vaccines are now eligible for global use and for procurement by Gavi, the Vaccine Alliance and UNICEF. These are the human monovalent (RV1) Rotarix™ (GlaxoSmithKline); human pentavalent (RV5) RotaTeq™ (Merck and Co., Inc); ROTAVAC^®^ (Bharat Biotech); and ROTASIIL^®^ (Serum Institute of India) (15–17). RotaTeq™ include human VP7 (G1-G4) and VP4 (P[8]) genotypes, Rotarix™ consists of a single human G1P[8] strain, whereas, Rotavac^®^ is a monovalent human-bovine RV vaccine containing genotype (G9P[11]) and RotaSIIL^®^ is a live attenuated human-bovine reassortant pentavalent RV vaccine containing VP7 genotypes (G1, G2,G3, G4, and G9) and VP4 genotype (P[5]) of bovine origin (17, 18).

In 2009, WHO recommended that all countries, and especially those with high child mortality rates for diarrhea, should implement rotavirus vaccines into their local immunization plans (19). By April 2020, 107 countries, of which 46 are eligible for Gavi, the Vaccine Alliance, have included rotavirus vaccines in their national or regional vaccination plans (20). The effectiveness of rotavirus vaccines is higher in developed, high-income countries (HICs) than in low income countries (LICs) and lower middle income countries (LMICs) (19, 21). Complicating factors such as nutritional status, age at infection, size of inoculum of infectious agent and interference by other enteropathogens may also impact on vaccine success in developing countries which suffer the greatest burden of rotavirus disease (22). Two additional rotavirus vaccines are available regionally, i.e., Rotavin-M1 in Vietnam and Lanzhou Lamb Rotavirus (LLR) in China (23, 24).

As rotavirus is continually altering its genetic makeup, it is therefore imperative to study how immunity derived from vaccines influences the development and spread of the vaccine-targeted major RVA strains (25). Vaccines that fail to eliminate a viral pathogen can apply pressures that cause changes in a microbial population’s genetic composition (26). Close human animal encounters and rise in global migration can transmit new virus virulence factors or mutants into unaffected populations, giving these viruses an opportunity to gain biological competitiveness against the common wild-type strains existing in the population (11). Such variations can be detrimental to the efficacy of the vaccine. Although immune responses to vaccines may be affected by several factors, human genetic differences are thought to have a significant effect on vaccine response variability (27). In defined virus epitopes, positive selection of single amino acid mutations gradually generates diversity of the viruses (28). The vaccine virus will not trigger an immune response, identifies and eliminates the newly emerged virus. Thus ELISA or other neutralization studies performed for variant strains can be affected.

The Government of Pakistan, with support from GAVI, the Vaccine Alliance, included Rotarix™ vaccine in the EPI schedule in 2018 (7). The post-vaccination rotavirus studies regarding the effectiveness of RVA vaccines and their effects on the particular genotypes of the country have yet to be identified (29). Over the pre-vaccine age, surveillance data from Pakistan have shown that G1P[8] is one of the most common RVA genotypes circulating in Pakistan over the last decade (30, 31). G1P[8] strains are responsible for the majority of human RVA infections (32). The main objective of this study was therefore to investigate the disparity between the circulating wild G1P[8] strains collected prior to vaccine implementation in Pakistan with the Rotarix™ and RotaTeq™ strains. The findings of this study will lead to the potential establishment of the pre-vaccination background for comparison studies after vaccination.

## Material and Method

### Study Samples

In the current study we selected six representative RVA G1P[8] strains based on the partial sequencing, phylogenetic analysis and their distinct circulating lineages. These specimens were already genotyped by partial sequencing in a hospital-based RVA surveillance study performed between January 2015 and December 2016 (7). The research population included children <10 years of age admitted with acute gastroenteritis in two hospitals in Rawalpindi, Islamabad, Pakistan, PIMS (Pakistan Institute of Medical Sciences and BBH (Benazir Bhutto Hospital). The complete sequences for six G1P[8] strains that were obtained in this study were needed for further comparative vaccine analysis.

### RNA Extraction

Fecal suspensions (10%) were prepared by adding 100 mg of fecal sample in 1 ml of phosphate buffer saline (PBS) in a sterile eppendorf tube. In compliance with the manufacturer’s instructions, a QIAamp viral RNA minikit (Qiagen) was used to extract viral RNA from the diluted stool content.

### RT-PCR and Sequencing

Extracted RNA were denatured at 95°C for 2 min, and RT-PCR was carried out using a Qiagen one-Step RT-PCR kit (20 μl of H_2_O, 5 μl of Qiagen One step RT-PCR buffer, 5 μl of diluted RNA, 1.5 μl of forward primer (10 µM), 1.5 μl of reverse primer (10 µM) and 1 μl of RT-PCR enzyme mix. The full VP7 and VP4 genes (regions VP8* and VP5*) were amplified using the previously described primers (25, 33–35). RT-PCR was carried out with an initial RT step at 50°C for 30min; Taq polymerase activation was carried out at 95°C for 15 min, followed by 40 cycles of amplification (denaturation 45 s at 94°C, annealing at 45°C for 45 s for VP4 and at 50°C for 45 s for VP7, extension 1 min at 72°C and final extension at 72°C for 10 min). The PCR product was purified using the ExoSAP-IT™ clean-up kit (Thermofisher Scientific, USA) and sequenced using the BigDye Cycle Sequencing Kit (Applied Biosystems, USA). The sequencing was carried out using forward and reverse primers that were used for the RT-PCR. Following the initial sequencing reaction, an ethanol precipitation was carried out and the final product was loaded in the automated sequencer ABI PRISM 3130 (Applied Biosystems, USA). Furthermore, the primer-walking sequencing was conducted to cover the full sequence of segments VP7 and VP4 (36).

### Sequence and Phylogenetic Analysis

Sequencing files were processed using Chromas v2.6.5 (Technelysium, Australia) and multiple sequence alignments were performed *via* CLUSTALW in the MEGA 6.0 (37). The maximum likelihood trees were built using the Kimura-2-parameter model in MEGA 6. The amino acids and nucleotides differences were determined using P-distance model in MEGA 6.0. The structural analysis of VP7 (PBD 3FMG) and VP8 (PDB 1KQR) was carried out using UCSF Chimera-Molecular Modeling tool (38).

### Research Data Availability

The data sets created for this analysis are available with following accession numbers on GenBank: **(VP7)** MT381737-MT381742 and **(VP4)** MT381743-MT381748.

## Results

### Phylogenetic and Sequence Analysis

Phylogenetic trees were constructed on the basis of full-length sequencing of six Pakistani G1P[8] strains for the VP7 and VP4 gene segments. G1 component of our study strains clustered in two distinct lineages I and II on phylogenetic observation, while their counterpart P[8] clustered into lineages III and IV, respectively. Rotavirus strains used in the synthesis of Rotarix™ and RotaTeq™ belong to lineages II and III, respectively. We analyzed the difference between nucleotide and amino acid (a) in antigenic regions for our G1P[8] strains and two globally available vaccines (Rotarix™, RotaTeq™).

**Figure 1:**
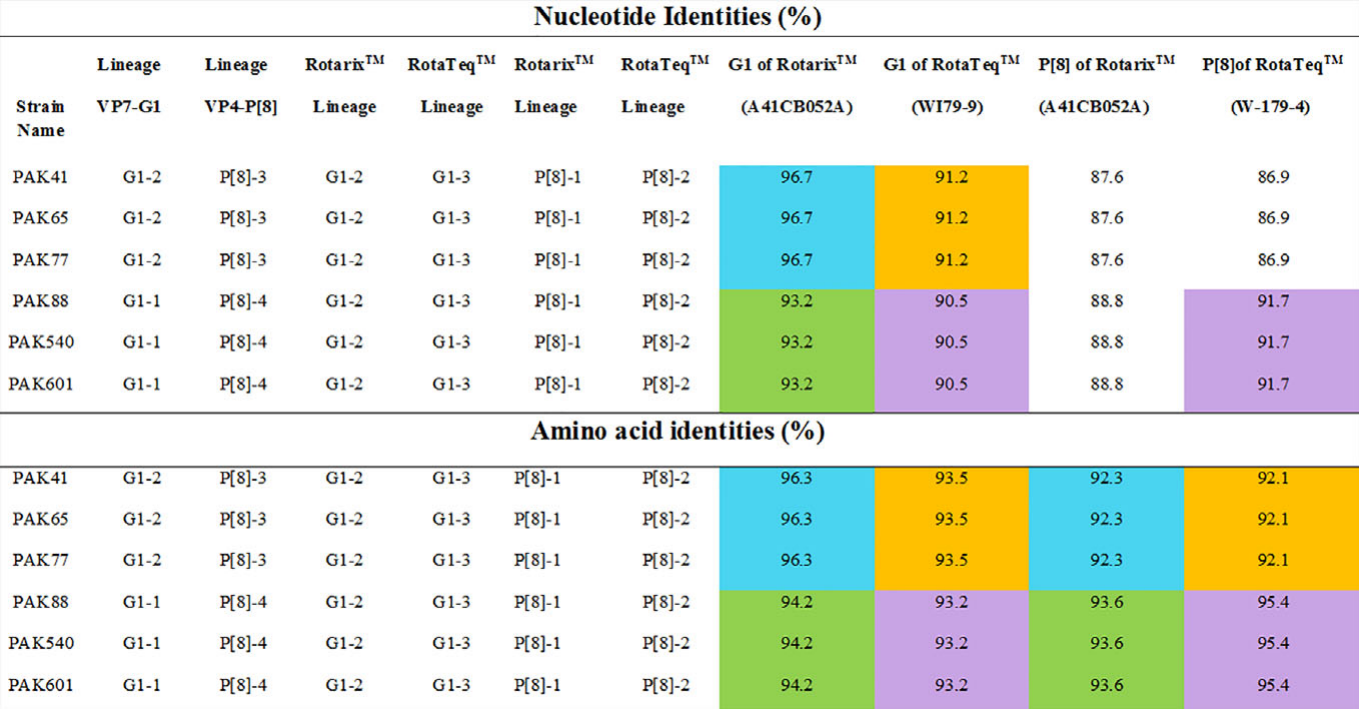
The nucleotides and amino acids differences of VP7 and VP4 proteins of Pakistani G1P[8] strains with rotavirus vaccine strains. The similarities between G1 lineage I to Rotarix™ and RotaTeq™ vaccine strains is shown in green and purple colours, respectively. While, the similarities between G1 lineage II to Rotarix™ and RotaTeq™ vaccine strains is shown in blue and orange colours, respectively.

**Figure 2:**
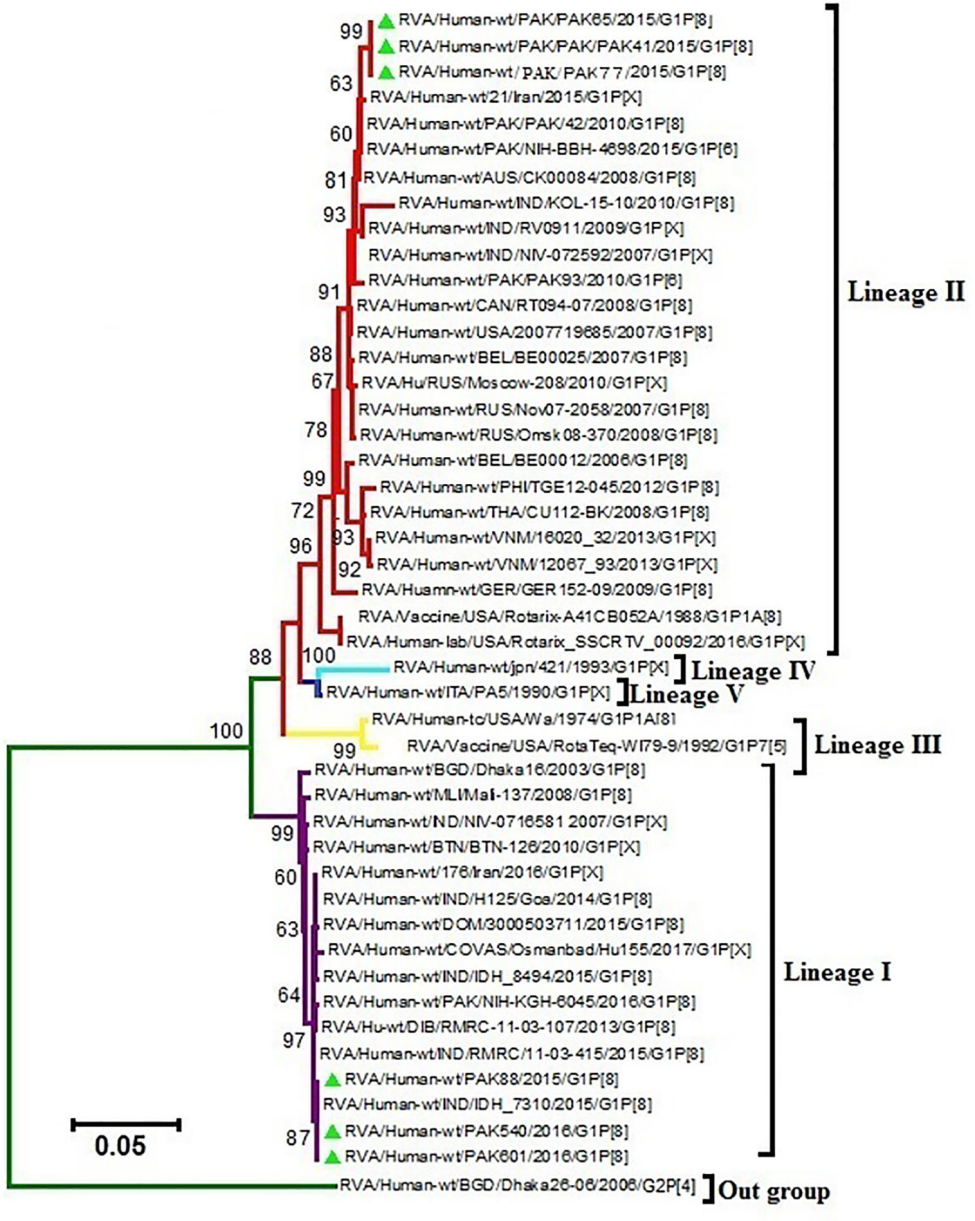
Maximum Likelihood tree of the VP7 protein of G1P[8] group A rotavirus (RVA) strains reported in Pakistan and the vaccine strains Rotarix™ and RotaTeq ™. Maximum Likelihood trees were generated using Kimura-2-parameter model in MEGA 6. Coloured branches represent different lineages. The lineage I is represented by purple colour branch including strains isolated from all over the world and our study strains represented by green triangles in the same branch. The lineage II is represented by red colour branch including strains isolated from all over the world and our study strains represented by green triangle in the same branch. Yellow colour branch includes strains belong to lineage III. Aqua colour branch includes strains belong to lineage IV. Blue colour branch includes strains belong to lineage V. G2P[4] RVA strain from Bangladesh is used as an out-group represented by green colour branch. Bootstrap replicates (1000) below than 60% are not shown in the tree.

Our study VP7 lineage II strains (PAK41, PAK65 and PAK77) showed nucleotide and amino acid identities of 96.7% and 96.3%, respectively to VP7 protein of Rotarix™ vaccine belong to lineage II. While, G1 lineage I strains (PAK88, PAK540 and PAK601) showed 93.2% nucleotide and 94.2% amino acid identities to that of Rotarix™ VP7 protein. On phylogenetic analysis these strains clustered closely with strain isolated from Iran (21/Iran), previously reported strains (PAK42 and NIH-BBH 4698)) from Pakistan and strains isolated from all around the world in VP7-G1 lineage II. Our study VP7 lineage I strains exhibited nucleotide and amino acid identities of 91.2% and 93.5%, respectively to VP7 protein of RotaTeq™ vaccine and showed 90.5% and 93.2% of nucleotide and amino acid identities, respectively to that of RotaTeq™ VP7 protein. On phylogenetic tree these strains showed close similarity with Indian G1 strains (IDH_73, DIB/RMRC-11-03-107, and DIB/RMRC-11-03-115), previously reported Pakistani strain (NIH-KGH-6045) and other worldwide reported strains in VP7-G1 lineage I (**Figures 1**, **2**).

**Figure 3:**
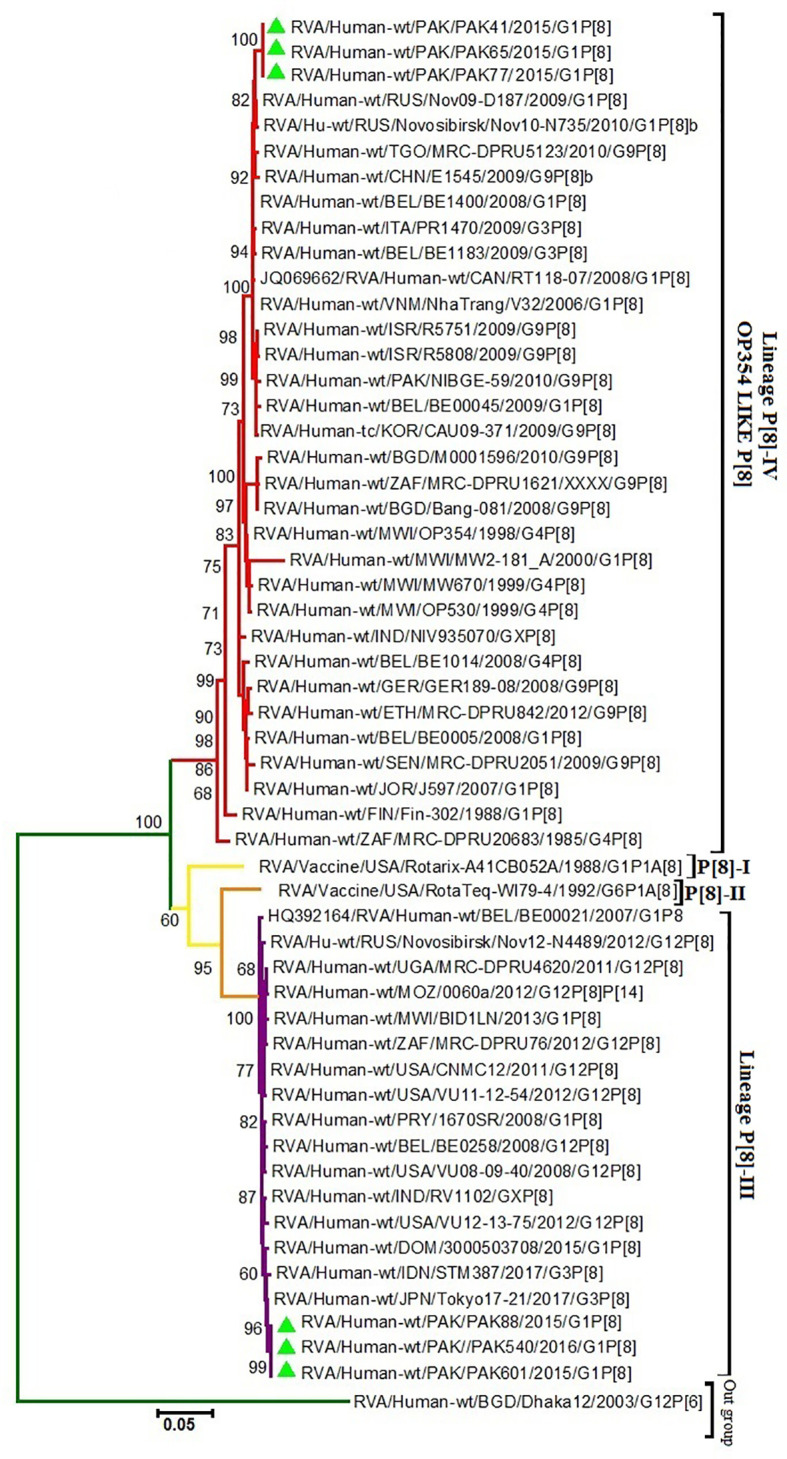
Maximum Likelihood Phylogenetic tree of the VP4 protein of G1P[8] Group A rotavirus (RVA) strains reported in Pakistan and the vaccine strains Rotarix™ and RotaTeq ™. Maximum Likelihood trees were generated using Kimura-2-parameter model in MEGA 6. Coloured branches represent different lineages. Yellow colour branch includes strains belong to lineage I and orange colour branch represent lineage II strains. The lineage III is represented by Purple colour branch including strains isolated from all over the world and our study strains represented by green triangles in the same branch. The lineage IV is represented by Red colour branch including strains isolated from all over the world and our study strains represented by green triangle in the same branch. RVA strain (G12P[6]) from Bangladesh is used as an as out-group is represented by green colour branch in the tree. Bootstrap replicates (1000) below than 60% are not shown in the tree.

VP4 of our study P[8] RVA strains that grouped into lineage IV were more closely related on amino acid and nucleotide level (95.4% and 91.7%, respectively) to VP4 protein of RotaTeq™ (W-179-4-lineage II) than lineage III of RotaTeq™ (W-179-9). Phylogenetic analysis have revealed that these strains clustered closely with strains isolated from, Africa, China, Belgium Russia (Nov09-D187, E1545, and BE1400) and with strains isolated from other countries of the world inVP4-P[8] lineage IV. VP4 of Pakistani P[8] RVA strains clustering in lineage III showed nucleotide and amino acid identities (87.6% and 92.3%, respectively) to VP4 protein of Rotarix™ (A41CB052A) and (86.9% and 92.1%, respectively) to the VP4 protein of RotaTeq ™ (W179-4). Phylogenetic study have revealed that these strains grouped together closely with strains reported in Japan Indonesia (Tokyo-17-21 and STM387) and other worldwide strains in VP4-P[8] lineage III (**Figures 1**–**3**).

### Comparative Analysis of VP7-G1 and VP4-P[8] Proteins of RVA Strains Circulating in Pakistan and Rotavirus Vaccines

In the current study, we have compared the VP7 and VP4 (VP8* and VP5*) antigenic epitopes of 6 Pakistani G1P[8] rotavirus strains to those of the RVA vaccine (RotaTeq ™ and Rotarix™) strains. The VP7 protein covers three vital antigenic regions (7-1a, 7-1b, 7-2). These regions contain 29 amino acid residues from position 87-291, based on strains found in rhesus and submitted with accession number AF295303 in GenBank (39).

**Figure 4:**
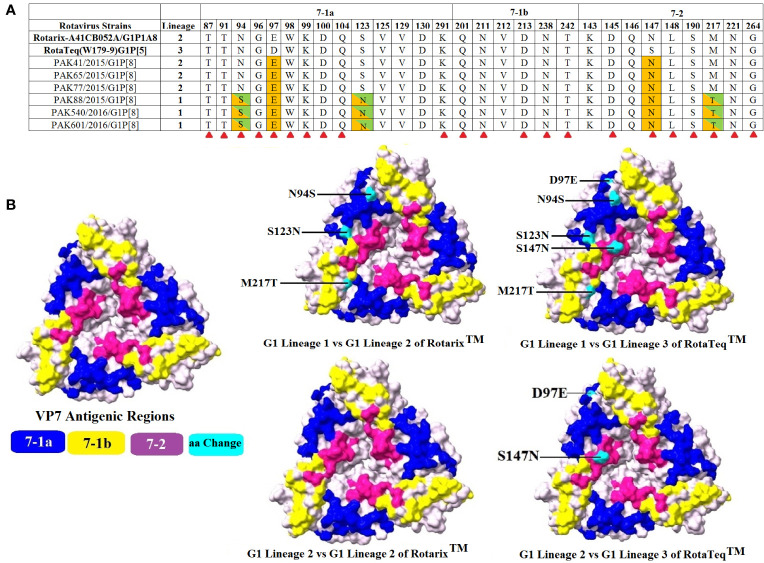
Alignment of the antigenic epitopes of VP7-G1 of the Pakistani group A rotavirus (RVAs) with those of Rotarix™ and RotaTeq™ vaccines. **(A)** The whole antigenic region is divided into three epitopes 7-1a, 7-1b, and 7-2. Amino acid residues highlighted in green/orange colours are those different from both Rotarix™ and RotaTeq ™, amino acid residues different from Rotarix™ and RotaTeq™ are marked with green and orange colours, respectively and amino acid residues believed to induce escape from the mAbs neutralization are marked with red triangle (40). **(B)** Surface representation of VP7 trimer (PDB 3FMG). Antigenic epitopes 7-1a, 7-1b, and 7-2 are marked in blue, yellow and purple colours, respectively. Cyan colour shows surface exposed residues which differ between our G1 strains and vaccine strains.

When our study G1 strains (lineage I and lineage II) were compared to two vaccines (RotaTeq™ and Rotarix™), the region 7-1b was highly conserved for both vaccines but differences were observed in two regions (7-1a and 7-1b). Comparing G1-lineage I strains with Rotarix™ G1P[8] strain, amino acid changes were observed at 2 positions in region 7-1a and at a single position in region 7-2. When G1-lineage I strains were compared with RotaTeq™ G1P[5] vaccine strain, amino acid changes at 3 and 2 positions were observed in region 7-1a and 7-2, respectively. Comparing G1-lineage II strains with the vaccine strain Rotarix™ G1P[8], no single change in amino acid was observed. While, on comparison with RotaTeq™ G1P[5] vaccine strain, two amino acid changes were observed, 1 in region 7-1a and 1in region 7-2 (**Figure 4**).

VP4 protein contains a total of 776 amino acids and is activated by the proteolytic cleavage into two subunits, i.e., VP8* and VP5*. The VP8* subunit consists of four antigenic regions, i.e., 8-1-8-4, while the VP5* subunit contains five antigenic regions, i.e., 5-1-5-5, that overall contain 37 amino acids (41). Comparing VP8* antigenic epitopes with RotaTeq™ and Rotarix™ vaccines amino acid differences were observed in three regions with the exception of region 8-4 which was found to be conserved in both our study and vaccine strains. When comparing VP5* antigenic epitopes with RotaTeq™ and Rotarix™ vaccines, amino acid changes were observed in one region 5-1, while four regions were found to be conserved for both our study and vaccine strains.

**Figure 5:**
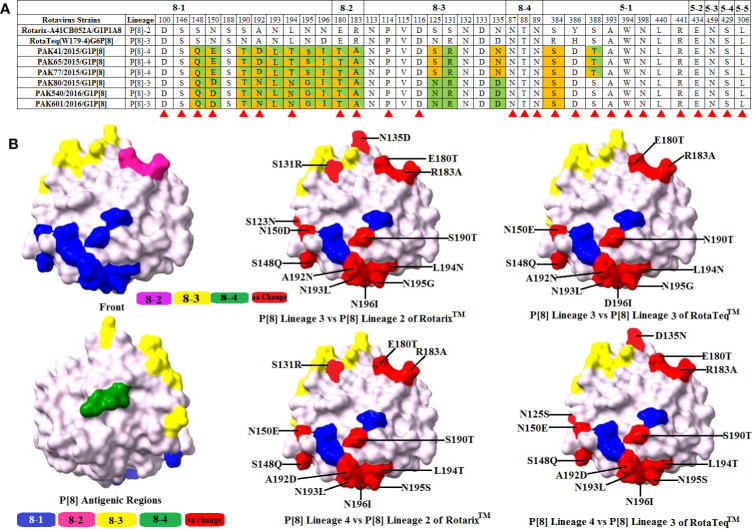
Alignment of the antigenic epitopes of VP4-P[8] of the Pakistani group A rotavirus (RVAs) with those of Rotarix™ and RotaTeq™ vaccines. **(A)** The whole antigenic region is divided into four epitopes 8-1, 8-2, 8-3, and 8-4 for VP8* and five epitopes for VP5*. Amino acid residues shading in green and orange are those different from Rotarix™ and RotaTeq™, respectively, amino acid residues highlighting in green/orange colour are those different from both Rotarix™ and RotaTeq™ and amino acid residues believed to induce escape from the mAbs neutralization are marked with red triangle (41). **(B)** Representation of surface exposed amino acid residues of VP4 core VP8* core (PDB 1KQR). Antigenic epitopes 8-1, 8-2, 8-3, and 8-4 are represented by blue, pink, yellow, and green colours, respectively. Red colour shows surface exposed residues which differ between our P[8] strains and vaccine strains.

When our study P[8]-3 strains were compared with Rotarix™ G1P[8] and RotaTeq™ G6P[8] vaccine strains, amino acids changes were observed at 14 and 12 positions, respectively. For Rotarix ™, changes were observed at positions 8, 2, 3, and 1 in regions 8-1, 8-2, and 8-3 and 5-1, respectively. For RotaTeq™, changes were observed at positions 8, 2, and 2 in regions 8-1 and 8-2 and 5-1, respectively. When our study P[8]-4 strains were compared with Rotarix™ G1P[8] and RotaTeq™ G6P[8] vaccine strains, amino acid substitutions were observed at 14 and 12 positions, respectively. For Rotarix™, in regions 8-1, 8-2, 8-3, and 5-1 amino acid changes were observed at 8, 2, 2, and 2 positions, respectively. For RotaTeq™, in regions 8-1, 8-2, 8-3, and 5-1 amino acid changes were observed at 8, 2, 1, and 1 positions, respectively. Overall, most of the changes detected in the Pakistani RVA viruses were identified in the VP4 head part (VP8*) and very few changes were observed in the body of the spike or VP5* (**Figure 5**).

## Discussion

Rotavirus gastroenteritis accounts for significant mortality rates in children under 5 years of age (42). The World Health Organization (WHO) has prioritised the development and introduction of vaccines to manage this disease due to the large global burden of rotavirus (43). Two Rotarix™ and RotaTeq™ RV vaccines recently implemented in children’s immunisation programs in many countries around the world are highly successful in helping to prevent RV infections (23). Both vaccines have been shown to be successful against commonly circulating genotypes of RVs including G1-4, G9, and P[4], P[6], and P[8] (25). However, as a result of the long-term use of such vaccines, possible vaccine escape strains could be selected in the future (44, 45).

In the current study, we compared antigenic differences between VP7-G1 and VP4-P8 (VP8*, VP5*) proteins of circulating human RVA strains in Pakistan and two available vaccines (RotaTeq™ and Rotarix™). Of the two lineages (lineage I and II) of VP7-G1 strains circulating in Pakistan, lineage I showed a few amino acids variations from both RotaTeq™ and Rotarix™ belong to lineages II and III, respectively. Pakistani G1-lineage II strains, however, showed remarkable similarities to the Rotarix™ vaccine strain.

RV glycosylation increases their pathogenicity and enhances their resistance to mAbs (46). Two N-linked glycosylation sites at positions 69 and 238 have been identified in the G1 genotype of VP7 protein vaccine strains, which were found to be conserved in the Pakistani RVA G1P[8] strains. Amino acid substitution S123N, N94S, and M217 T have been observed between strains of G1- lineage I. The rotavirus vaccine was introduced in Pakistan in 2018. It was initially proposed that the strains found in 2015–2016 may occur in our natural population. However, these variations were also reported between the G1P[8] strain in Australia, Belgium, Labenon, Russia and India (25, 33, 47–49). It was suggested, therefore, that they could have been introduced into Pakistan from other countries of the world.

In this study, strains of G1 lineage I are paired with lineage IV of P[8] and G1 lineage II with P[8] lineage III. In the previous studies reported in Belgium and India, lineage I strains are coupled with lineage III of P[8] and lineage II of G1 paired with lineage IV of P[8] (33, 47). Thus, a full genome analysis of these strains is proposed in the future that may better clarify these genotypic interchanges between the different G1P[8] strains.

Our study’s P[8] lineage III and IV genotypes are quite different from the lineages I and II, respectively, of both Rotarix™ and RotaTeq™. The lineage IV strains are also called OP354-like or P[8]-b. These OP354-like strains have recently gained interest because these are being detected all over the world after being first reported in Malawi (50). These findings are supported by phylogenetic analysis, where lineage III and IV strains clustered away from vaccine strains and showed close relationships with previously reported RVA strains from Japan and Russia, respectively.

Significant variations in the genes of VP7 may impair immunity, resulting in ineffective results or vaccine response failure. In contrast with the G types, poor immune responses to P[8] strains can be compensated for due to the relative lack of diversity between P-types (25). It has been shown that the immune response to the VP4 antigen is important and there are cross-reactive epitopes on the VP4 protein. Compared with the G types, the relative lack of diversity among P-types may contribute to heterotypic immune response (51).

Studies from Europe and America showed that variations in the sequence of VP7 and VP4 genes of circulating RVA strains and vaccine strains in these countries had no effect on the efficacy of the vaccines (47). In fact, Rotarix™ a monovalent rotavirus vaccine is believed to be effective against non G1P[8] rotavirus strains (52). The conserved immunogenic regions in VP7 and VP4 genotypes and non-structural proteins or internal proteins seems to be the reason for this cross protection (18). Earlier studies have shown that primary rotavirus infections are associated with rises in serum antibody levels to the structural proteins VP2, VP4, VP6, VP7, and to the non-structural proteins NSP2 and NSP4 (53, 54).

Since both vaccines have shown considerable efficacy to date in Europe and America, however, their long-term use has been suggested to contribute to the selection of strains capable of avoiding vaccine-induced immunity (44). However, after the introduction of Rotarix™ in Belgium, the incidence of Rotarix-like G1P[8] strains has declined more than the G1P[8] strains belonging to G1 and P[8] lineages that are less similar to Rotarix™ (33).

Overall, the results of the current study showed potential antigenic disparities in circulating G1P[8] strains prior to the RV vaccines introduction in Pakistan compared to Rotarix™ and RotaTeq™ vaccines. The findings of this study will provide baseline information for a post-vaccine study to gain insight into the effect of the introduction of the vaccines on the diversity of the rotavirus genotypes in Pakistan with respect to their subgenotypic lineages. But the exact effect of differences in amino acids in antigenic epitopes cannot be predicted from sequence and structural information alone. We believe that the findings of this research will be strengthened by continuous surveillance of circulating RVAs in line with increasing sample size and vaccine matching evaluation using genotype-specific antibodies and neutralization assays.

## Data Availability Statement

The datasets presented in this study can be found in online repositories. The names of the repository/repositories and accession number(s) can be found in the article.

## Ethics Statement

The studies involving human participants were reviewed and approved by Ethical Committee of PIMS and Benazir Bhutto Hospitals and Internal Review Board of COMSATS University, Islamabad, Pakistan. Written informed consent to participate in this study was provided by the participants’ legal guardian/next of kin.

## Author Contributions

AS (COMSATS University, Islamabad, Pakistan) planned the project and carried out experiments, data analysis and composed article. NB (COMSATS University, Pakistan) created idea and helped to start writing the article. All authors contributed to the article and approved the submitted version.

## Funding

This study was funded by Pakistan’s Higher Education Commission (HEC), Grant number (2BM1-196) as part of a Doctorate programme.

## Conflict of Interest

The authors declare that the research was conducted in the absence of any commercial or financial relationships that could be construed as a potential conflict of interest.
