# Corrigendum: Comparative Analysis of G1P[8] Rotaviruses Identified Prior to Vaccine Implementation in Pakistan With Rotarix™ and RotaTeq™ Vaccine Strains

**DOI:** 10.3389/fimmu.2020.625157

**Published:** 2020-12-16

**Authors:** Asma Sadiq, Nazish Bostan

**Affiliations:** Department of Biosciences, COMSATS University (CUI), Islamabad, Pakistan

**Keywords:** G1P[8], rotavirus, genotype, antigenic epitopes, emergence

In the original article, there was an error in the correspondence section. The only corresponding author is Nazish Bostan, nazishbostan@comsats.edu.pk. A correction has therefore been made to the correspondence section and should read as follows:

*Correspondence: Nazish Bostan, nazishbostan@comsats.edu.pk

The authors apologize for this error and state that this does not change the scientific conclusions of the article in any way.

